# The experiences of women diagnosed with uterine fibroids in the Kingdom of Eswatini

**DOI:** 10.4102/hsag.v29i0.2724

**Published:** 2024-11-15

**Authors:** Vuyisile J. Ginindza, Fortunate S. Shabalala, Bonisile S. Nsibandze, Makandwe Nyirenda, Themba G. Ginindza

**Affiliations:** 1 College of Health Sciences, School of Nursing and Public Health, University of KwaZulu-Natal, Durban, South Africa; 2 Department of Community Health Nursing, Faculty of Health Sciences, University of Eswatini, Mbabane, Eswatini; 3 Department of General Nursing, Faculty of Health Sciences, University of Eswatini, Mbabane, Eswatini; 4 Discipline of Public Health, School of Nursing and Public Health, University of KwaZulu-Natal, Durban, South Africa; 5 Burden of Disease Research Unit, South African Medical Research Council, Cape Town, South Africa

**Keywords:** uterine fibroids, Eswatini, experiences, women, symptoms, disease

## Abstract

**Background:**

Uterine fibroids (UFs) are benign uterine growths that significantly impact women’s daily activities, quality of life, fertility and expenditure.

**Aim:**

This study aimed to provide in-depth insights into the lived experiences of women diagnosed with UFs.

**Setting:**

The study was conducted in Eswatini health facilities across the four geographic regions.

**Methods:**

An explorative qualitative design was used; participants with confirmed UFs diagnoses or surgery related to UFs were purposively recruited for focus group discussions. Data collection was guided by the revised Wilson and Cleary model of health-related quality of life (HRQOL), which was iteratively analysed using Braun and Clark’s thematic analysis framework.

**Results:**

Sixty participants with confirmed UFs diagnoses or surgery related to UFs were included in this study. Five themes emerged: awareness of the disease, symptoms experienced, treatment and care, perceptions and beliefs and health-seeking behaviour. Most participants experienced physical symptoms, abdominal pain and vaginal bleeding. Among the emotional and psychological consequences experienced by the majority of participants were fear, worry, tension and (perceived) disrupted body image. The majority of the participants lacked knowledge of UFs, and their perceptions and health-seeking behaviour differed.

**Conclusion:**

Findings showed that most participants experienced physical, psychological, emotional and social challenges.

**Contribution:**

These experiences were influenced by lack of knowledge, symptoms experienced, poor treatment and care and perceptions and beliefs about UFs. Health education, client involvement, early diagnosis and effective treatment are recommended to improve the experiences of UFs.

## Introduction

Uterine fibroids (UFs) are a prevalent concern for women’s sexual and reproductive health worldwide. These non-cancerous tumours cause major distress and negatively impact the quality of life for affected women (Navarro et al. 2021). Symptoms commonly include heavy bleeding, bladder dysfunction and pregnancy complications (Marsh et al. 2018). The prevalence of UFs in the sub-Saharan Africa (SSA) region, including Eswatini, is high but not well determined (Morhason-Bello & Adebamowo 2022; Sefah et al. 2023). Varying prevalence rates have been reported in different countries, ranging from 20% in Kenya to 36% in Ghana (Adawe et al., 2022). Despite these numbers, there are limited data on overall patient experiences with UFs in SSA, and in Eswatini, no previous studies have been conducted on UFs. The purpose of this study was to explore the lived experiences of women diagnosed with UFs. The findings will be used to inform the Sexual Reproductive Health (SRH) Programme to develop effective UFs’ management and care strategies for women’s overall well-being.

Findings from another UFs research study identified that women diagnosed with UFs face multiple problems including heavy bleeding, painful menstruation, painful intercourse, pelvic pain and pregnancy complications (Freytag et al. 2021). The symptoms of UFs, such as pelvic pain, and heavy bleeding can significantly impact women’s lives, causing unproductivity, absenteeism and impaired fertility (Brito et al. 2014). Uterine fibroids have also been identified as a leading cause of infertility; a study conducted in Côte d’Ivoire found that 41.8% of the population studied experienced infertility because of Ufs (Dia et al. 2017). Women with symptomatic UFs, particularly those under 40 years, expressed concerns about preserving fertility (Knudsen et al. 2017). Moreover, women with submucosal fibroids had lower rates of pregnancy and live births (Freytag et al. 2021). Uterine fibroids harmed women’s livelihoods, resulting in fear and discouragement, and further affected their roles in both domestic and social contexts (Brito et al. 2014). Additionally, UFs can negatively affect the workplace environment and careers, with a significant number of employed women missing work, and not attaining career goals (Fortin et al. 2018). Uterine fibroids noticeably affect women psychologically, resulting in feelings of fear, worry and frustration before and after diagnosis (Ghant et al. 2015). Other identified factors that contributed to increased anxiety and worry among women with UFs were the physical symptoms, treatment options, complications and lack of adequate information (Knudsen et al. 2017). In consecutive studies, the same author noted that most women with UFs experienced high mental distress and anxiety because of fear associated with the disease (Knudsen et al. 2019). These psychological impacts highlight the need for psychological counselling alongside the physical management of UFs.

## Research methods and design

### Study setting

This study was conducted in all four geographic regions of the Kingdom of Eswatini between August 2021 and December 2022. A total of eight admitting hospitals were purposely selected. The hospitals were a mix of public, private, mission owned and in both urban and rural locations. They included four government hospitals, two private hospitals and two mission hospitals.

### Population

Sixty women were selected for focus group discussions. The inclusion criteria were women aged between 25 and 64 years to explore views of women in the different life stages, reproductive, menopausal and post-menopausal stages. The participants must have a confirmed UFs diagnosis or had any surgical procedure related to UFs (hysterectomy, myomectomy, laparoscopic or uterine embolisation), participated in our primary UFs study and voluntarily consented to participate in focus group discussions. Exclusion criteria were being younger than 25 or over 64 years, not having a confirmed UFs diagnosis or UFs-related surgery, not participating in the primary study or refusing to participate in focus group discussions. Measures to ensure active group participation were considered, including briefing participants on the purpose of the study, and ethical principles, emphasising the importance of respecting each other’s viewpoints. The presence of a facilitator who guided the discussions, the purposive sampling method and focus group sizes effectively ensured a balanced distribution of different age groups and promoted active participation among all.

### Study design

An explorative qualitative study design was conducted using focus group discussions to enable participants to share their experiences with UFs. Focus group discussions were chosen because they promote diversity of opinion as many participants voice their ideas. They also aid in improving the participants’ and researcher’s comprehension of the subject under discussion. Additionally, focus group discussions help participants learn and reflect more, facilitating an evaluative narrative creation. (O. Nyumba et al., 2018).

### Sampling and sample size

Sixty women with a confirmed diagnosis of UFs or those who had surgery related to UFs were purposively recruited. Each focus group had six to eight participants.

### Study instruments and data collection

Participants were recruited during their Outpatient Department appointments or ward admissions. The focus group discussions were conducted by the principal investigator and two research assistants per group, who were degreed nurse midwives working in the department. They were well trained in conducting focus group discussions and understood the topic and research ethical considerations. Six groups had eight participants, and the other two groups had six participants. The discussions were conducted in the Siswati language, translated into English and lasted for 45 minutes. An interview guide and probes were used to collect data, and field notes were taken by the research assistants. Data were audio recorded during the group discussions and transcribed verbatim by the principal investigator. Initially, five domains were discussed, adopted from the revised Wilson and Cleary model of HRQOL: (1) biological status, (2) symptoms, (3) functional health, (4) general health perceptions and (5) overall quality of life.

### Data analysis

Discussions around the five domains were audio recorded and transcribed verbatim. An inductive thematic analysis was conducted to identify themes using Braun and Clarke’s steps. A total of five themes emerged directly from the data: awareness of the disease, symptoms experienced, treatment and care, perceptions and beliefs and health-seeking behaviour. The data from the focus group discussions were also evaluated to determine data saturation, which is when further interviews were unlikely to provide new information. The audio-recorded data were transcribed by the principal investigator and were then independently reviewed by the two research assistants who were present during the focus group discussions for quality assurance and to ensure that information was consistent with the recalled conversations, field notes and observations. The transcripts were read repeatedly, interpretively and reflexively for better understanding. Initial coding was done independently by the research team, who later met to review and refine the codes for quality purposes. Themes and subthemes were then developed independently by the research team, who later met to discuss the themes with regular reference to the transcripts to ensure that the suppositions corresponded with the participants’ submissions. The themes were collaboratively discussed until a consensus with five themes was reached. To ensure confidentiality, pseudonyms were used, and other identifiers were omitted.

**Rigour:** Several strategies were adopted to ensure the methodical rigour and trustworthiness of the study.

**Credibility:** The data from the focus group discussions were transcribed verbatim within 24 hours by the principal investigator and two research assistants. The team members also engaged with the data independently, reviewing the transcripts and audio recordings in an iterative process to ensure the credibility of the findings. Themes were rooted in the data with continuous reference to the audio and transcripts.

**Dependability:** Throughout the study, there was a detailed record of data collection methods. The study protocol was communicated to both research assistants and participants before and during the study. The team regularly met to discuss the analysis process and reached a consensus for reliability purposes.

**Confirmability:** During the data collection process, triangulation was used, which involved gathering data from various sources including recorded audio, observations and notes taken during discussions. To ensure reflexivity, themes were collaboratively discussed until a consensus was reached. Results were supported with illustrative quotes from participants, which were written verbatim with minor edits to improve clarity.

**Transferability:** The purposive sampling method was utilised to ensure accurate participants were chosen for the study. Focus groups were distributed throughout the country to provide fair representation. Participants were confirmed to have UFs diagnosis.

### Ethical considerations

Ethical approval was obtained from the University of KwaZulu-Natal Biomedical Research Ethics Committee (BREC), BREC/00002571/2021 and the Eswatini Health and Human Research Review Board (EHHRRB), EHHRRB023/2021. Permission was obtained from the proposed research sites (gatekeepers), regional health administrators, chief executive officers, matrons and nurse managers. Participation in the research study was voluntary. Individuals selected for the focus group discussions were taken through an informed consent process that included information on the research purpose and focus group discussion procedures. All participants individually consented and signed a written consent form. Participants were assigned pseudo-names to ensure confidentiality (UF4, participant 3).

## Results

### Participants’ characteristics

Sixty women who met the inclusion criteria participated in the study. A majority of the participants were aged 35–39 years, married, had tertiary education and had undergone myomectomy for UFs. The details of the participants’ demographic characteristics are provided in Table 1.

**TABLE 1:** Demographic characteristic of the focus group discussion participants (*N* = 60).

Demographics	*n*
**Age (years)**	
25–29	5
30–34	8
35–39	15
40–44	9
45–49	10
50 and above	13
**Marital status**	
Not in a relationship	8
In a relationship	15
Married	37
**Educational level**	
Primary/illiterate	6
Secondary	13
O’level	15
Tertiary	26
**Regions of Eswatini**	
Hhohho	22
Manzini	22
Lubombo	8
Shiselweni	8
**Types of gynaecological diagnosis**	
Uterine fibroids without surgery	21
Myomectomy	27
Hysterectomy	12

### Themes

The participants’ experiences were categorised into 5 themes and 15 subthemes as indicated in Table 2. The themes are awareness of the disease, symptoms experienced, treatment and care, perceptions and beliefs and health-seeking behaviours. Themes emerged from using the inductive thematic analysis of the data.

**TABLE 2:** Themes and subthemes that were identified from the focus group discussions.

Themes	Subthemes
1. Awareness of the disease	1.1. Lack of knowledge 1.2. Lack of uterine fibroid education from health clinicians 1.3. Myths and misconceptions
2. Symptoms experienced	2.1. Physical symptoms 2.2. Psychological symptoms 2.3. Emotional symptoms 2.4. Social symptoms
3. Treatment and care	3.1. Dissatisfactions with current treatment 3.2. Limited involvement in the plan of care
4. Perceptions and beliefs	4.1. Perceived as demonic or a curse 4.2. Belief in traditional medicine cure 4.3. Perceived as cancer-related
5. Health-seeking behaviour	5.1. Not getting better from other healing systems 5.2. Presenting unusual and unbearable symptoms 5.3. Fear of the disease outcomes

#### Theme 1: Awareness of the disease

**Subtheme 1.1: Lack of knowledge:** Participants were not aware of UFs as a disease. Even when they experienced the symptoms, they did not know the disease symptoms or the treatment options of UFs. Some participants shared this:

‘I was in a lot of pain and bleeding. I had this problem for some time, but I did not know what was wrong with me.’ (UF1, participant 3, 40 years, not in a relationship)

‘I had no idea how the disease was treated so when the Doctor told me that I needed surgery, I was surprised and asked him for more details of the disease treatment options.’ (UF3, participant 4, 43 years, married)

**Subtheme 1.2: Lack of uterine fibroi d education from health clinicians:** There is limited information about the disease; participants got information from the internet, friends or other patients who had the disease so they expressed the need for increased public awareness and education about UFs:

‘In the hospitals, there is no explanation of the disease process; it is like they are not sure; I wish the health professionals could provide more information because even the internet is not helping much, and we are not sure if we are getting the right information.’ (UF4, participant 3, 60 years, not in a relationship)

‘I depend on the internet for any information about the disease because there is no detailed information in the hospital and this worries me because not everything is true on the internet.’ (UF1, participant 4, 26 years, in a relationship)

**Subtheme 1.3: Myths and misconceptions:** Most of the participants had different myths and misconceptions about UFs. Following are some of the views shared by participants:

‘It is a disease that eats your blood in the body, and then you cannot breathe properly.’ (UF7, participant 1, 45 years, married)

‘I think it is a disease caused by the remains of menstrual blood in the uterus.’ (UF4, participant 1, 62 years, not in a relationship)

#### Theme 2: Symptoms experienced

The participants experienced various symptoms that affected of all dimensions of daily life causing significant physical, emotional, social and psychological burden of the disease.

**Subtheme 2.1: Physical symptoms:** Participants experienced uncomfortable physical symptoms such as pelvic pain, abnormal vaginal bleeding, abdominal distension, sexual dysfunction and urological symptoms.

Some participants shared the following:

‘I experience severe lower abdominal pains, scanty irregular menstruation, and difficulty passing urine even if I have the urge to pass urine, it does not come out. I think it is because the doctors have delayed removing the fibroids.’ (UF1, participant 2, 58 years, married)

‘My menstruation is very irregular with severe lower abdominal pain and heavy bleeding which sometimes last for ten days.’ (UF2, participant 3, 37 years, married)

Some participants expressed concern over self-image, attributing it to the massive weight gain around the waist as shared by one of the participants:

‘Another thing is that the abdomen is growing; when people see you, they even say you are pregnant, I don’t explain anything to them anymore. It changes your body shape.’ (UF7, participant 3, 36 years, in a relationship)

Most participants were also worried about the decline in sexual activity. Some participants shared their experiences as follows:

‘We now take days before we can enjoy sex because it is painful; it is like the fibroids have moved down to the vagina, making penetration painful and difficult.’ (UF4, participant 6, 41 years, married)

‘The heavy bleeding and pelvic pain deprive us of sexual activity.’ (UF5, participant 2, 40 years, married)

**Subtheme 2.2: Psychological symptoms:** Most participants experienced overwhelming psychological burden because of UFs complications and their treatment modalities. They experienced worry, anxiety and depression because of the recurrence of the UFs and treatment options. Other participants shared the following psychological impact of UFs:

‘I do not want to be touched or examined by any gynaecologist because it depresses me to be told you have uterine fibroids again, and you must remove them. The other time, I just walked away and left the doctor.’ (UF7, participant 2, 41 years, in a relationship)

‘The surgery itself causes anxiety. Is there any better way that we can treat the uterine fibroids?’ (UF1, participant 6, 30 years, married)

Some participants experienced stress, frustration and conflicts within families because of infertility caused by UFs as highlighted in the following quote:

‘There is no peace when there is no child; it hurts so much, especially when you hear others sharing about how they bond with their babies during breastfeeding; I just walk away from such discussions and cry.’ (UF5, participant 3, 33 years, married)

‘I am very depressed and have given up on having a baby, am 40 years old now, had uterine fibroids and removed them but later they reoccurred. The doctor has advised that I must have a hysterectomy but I am not ready for that.’ (UF7, participant 4, 40 years, in a relationship)

**Subtheme 2.3: Emotional symptoms:** The participants also experienced emotional distress, fear of treatment options and outcomes, UFs symptoms, inability to conceive, rejection and isolation and often avoiding public spaces. Some of the participants shared the following emotional burden of the disease:

‘On my menstrual days, the bleeding becomes heavy. I will even be scared to walk around people, I will not even attend church services, and I just stay at home fearing I might mess- up myself. Even at home, I will be covering my bottom with small blankets.’ (UF1, participant 1, 50 years, not in a relationship)

‘The way the disease is treated is scary; especially the surgery. I kept on wondering how life would be after surgery because initially I was done myomectomy then years later the UFs reoccurred and I went for hysterectomy.’ (UF4, participant 2, 49 years, married)

They also expressed secrecy around the condition and future effects of fibroid treatment. Many had difficulty sharing their diagnosis with others, friends or spouses, especially if a hysterectomy was done, mainly because they feared rejection. This was shared by some of the participants:

‘Honestly speaking having this disease is a challenge, it brings fear and shame. It is impossible to share this diagnosis, especially with friends and spouses because really who can continue a relationship with someone who has no uterus? You die with this information.’ (UF8, participant 1, 31 years, in a relationship)

‘I am scared to tell my husband that the Doctor has suggested that I do a hysterectomy because the UFs have reoccurred. I am scared I will lose him since I cannot have a baby.’ (UF5, participant 3, 33 years, married)

**Subtheme 2.4: Social sym ptoms:** The participants experienced negative social impacts such as loss of marriage prospects because of infertility, poor relationships and mistrust between couples related to sexual disturbances and self-isolation. Some participants shared their different experiences as follows:

‘Our male partners always blame us or end the relationship when we fail to conceive so, I think they must be involved when we are diagnosed with uterine fibroids to avoid blaming each other when there are no children or when the other partner is sick.’ (UF7, participant 3, 36 years, in a relationship)

‘Uterine fibroids cause pain and discomfort during sexual intercourse, which caused me to avoid sex. My husband thought I was cheating on him, and that hurt me because I thought if I didn’t have sex with him, he would get satisfaction from other girls and leave me.’ (UF2, participant 4, 37 years, married)

#### Theme 3: Treatment and care

Most participants expressed dissatisfaction with the current treatment options, scepticism about pharmaceutical outcomes, fear of complications and concern about delayed diagnosis and surgery. Participants further stated that the number of women presenting to the UFs clinic for management of the disease was higher.

**Subtheme 3.1: Dissatisfactions with current treatment:** The participants were disappointed and doubted the treatment modalities because of the worsening signs and symptoms even though they complied with pharmaceutical treatment.

Some participants had this to share:

‘I have to go back for review; if the doctor does not remove the uterus, it means I will take the contraceptives and ferrous sulphate for the rest of my life. Taking the contraceptives is very frustrating because I am not getting better and honestly why should I take them because I am not even sexually active? The ferrous sulphate makes the fibroids grow bigger.’ (UF1, participant 1, 50 years, not in a relationship)

‘We need better management of the disease other than the myomectomy because I did this procedure and years later the UFs reoccur.’ (UF4, participant 2, 49 years, married)

**Subtheme 3.2: Limited involvement in the plan of care:** The participants were also not happy with being less involved in the plan of care; they felt their views and feeling did not matter and that the doctors made the final decision.

Following are quotes from participants that expressed the discontentment:

‘In the hospital, they take a long time before intervening, and they do not listen to our concerns: I was ready to remove the fibroids, but the Doctor told me, the fibroids will shrink, I should not worry yet I was in pain and the vaginal bleeding was worsening. I feel like the disease is given less attention, yet the disease is in a delicate place.’ (UF8, participant 1, 48 years, married)

‘No matter how much I changed hospitals and doctors, the doctors insisted that they cannot surgically remove the fibroids because of my age. No one considered that I was ready for the surgical procedure.’ (UF1, participant 2, 58 years, married)

#### Theme 4: Perceptions and beliefs

Participants of this study had varied perceptions and beliefs about UFs, which affected their symptom experience and response to the disease. Some of those who had done the hysterectomy believed it was the only solution to the disease because you would be relieved of most of the physical symptoms.

**Subtheme 4.1: Perceived as demonic or a curse:** Some participants believed UFs were of demonic origin; they had to fight the demons, and they preferred prophetic assistance in managing the disease. One participant shared her experience as indicated by the following quote:

‘A relative once asked that I advise her daughter who had UFs because she refused medical treatment. When I discussed this with her, she openly told me that her Prophet was praying for her and had given her something that broke down the UFs (“I have witnessed the UFs break down, so I believe I will be completely healed”).’ (UF4, participant 2, 49 years, married)

‘This is demonic, one moment you are fine then then the next thing you know, you are diagnosed with fibroids, and the cause is not clear.’ (UF8, participant 4, 32 years, in a relationship)

**Subtheme 4.2: Belief in traditional medicine cure:** Others strongly believed that only traditional medicine could treat UFs, so they prioritised the traditional management. Some participants shared their beliefs as stated in the following quote:

‘I know someone who used traditional medicine, but you must start early. It breaks down the fibroids. I was late to start the traditional medicine; my fibroids were 7cm, but they were reduced to 5cm after I used the traditional medicine; they did not completely break down; only a bit broke down. The medication from the hospital does not help us. Instead, it makes the fibroids bigger.’ (UF7, participant 1, 45 years, married)

‘The traditional medicine is effective in the treatment of fibroids, I actually used it and it worked.’ (UF7, participant 1, 45 years, married)

**Subtheme 4.3: Perceived as cancer-related:** Participants expressed fear and worry when the UFs were not removed. They believed it could cause cancer. Another participant highlighted her concerns as indicated by the following quotes:

‘I do not understand why the Doctors are refusing to remove this fibroid, I have been to public and private hospitals without help, and I am scared that the more they delay removing it, it will progress to cancer.’ (UF6, participant 1, 60 years, not in a relationship)

‘I believe this will result in cancer because the doctors are delaying to remove the fibroids.’ (UF1, participant 2, 58 years, married)

#### Theme 5: Health-seeking behaviour

Participants’ health-seeking behaviours were influenced by various factors including the physical, emotional and psychological impacts of UFs, their beliefs and perceptions and not getting better from other healing healing systems.

**Subtheme 5.1: Not getting better from other healing systems:** Some participants tried several non-medical options to treat UFs; however, when they all failed, they then opted for the medical and surgical treatment of UFs. Following are quotes shared by some of the participants:

‘I tried several expensive fibroids treatment products from friends, social media adverts and streets, but none worked. I eventually agreed to the uterus removal and I have never been sick again.’ (UF8, participant 4, 57 years, married)

‘I used the traditional medicine for some time but the fibroids were not all removed so the symptoms worsened and I eventually went back to the hospital for removal.’ (UF5, participant 1, 47 years, in a relationship)

**Subtheme 5.2: Presenting unusual and unbearable symptoms:** Other participants were driven by the impact of the physical symptoms to seek medical care. Most participants highlighted how they were driven by the severity of symptoms to seek medical help as mentioned in the following quotes:

‘I had long heavy menstrual bleeding which lasted for over 7 days, but I ignored it until the one day while in town I experienced severe dizziness, collapsed, and was rushed to the hospital, the Doctor told me I had uterine fibroids, 8cm big.’ (UF6, participant 3, 32 years, married)

‘I was so weak, very dizzy and my skin was yellow, it was then that I decided to go to hospital.’ (UF7, participant 1, 45 years, married)

**S ubtheme 5.3: Fear of the disease outcomes:** Some participants were primarily driven by the fear that UFs will progress to cancer and infertility as indicated in the following quotes:

‘I am even scared that the more the doctors delay removing the fibroids, I might develop cancer.’ (UF1, participant 2, 58 years, married)

‘I have heard that fibroids cause infertility, I wish they just remove the fibroids earlier.’ (UF4, participant 3, 34 years, married)

## Discussion

This study qualitatively investigated the experiences of women diagnosed with UFs in the Kingdom of Eswatini. The study revealed that participants experienced physical, psychological, emotional and social challenges that significantly affected their well-being and quality of life. The participants’ beliefs and perceptions about UFs influenced their health-seeking behaviour and tolerance of the symptoms experienced.

The study revealed that most participants had limited knowledge about UFs, which influenced their response to symptoms and health-seeking behaviour. Most participants highlighted the need for public education and increased awareness of the disease. The results from this study tie in with other findings from similar studies where a lack of knowledge about UFs was observed, and the need for increased awareness of the disease was identified (Aninye & Laitner 2021; Brito et al. 2014; Dykstra et al. 2023). The SRH programme needs to be informed to launch nationwide campaigns to eliminate misconceptions and fear. Health practitioners should prioritise individualised client education to improve informed decision-making and satisfaction. Increasing UFs awareness can address beliefs and attitudes that influence health-seeking behaviour and disease impact.

Most participants in this study experienced physical symptoms such as abnormal vaginal bleeding, lower abdominal pains, altered daily activities, interrupted sexual activity, poor self-image, infertility and anaemia. Previous studies suggested that most women with symptomatic UFs suffer pain and abnormal uterine bleeding (Fortin et al. 2018; Swain et al. 2019). Findings from this study also noted that UFs symptoms and treatment options may limit the performance of daily living, similar to findings from previous UFs research studies (Ebrahiem Elsaied et al. 2020; Hsieh, Lu & Liang 2021; Ming et al. 2019). Owing to the physical impact of UFs, some participants often missed work or church. These findings correspond to other research findings from previous studies, which identified that symptomatic UFs resulted in reduced work productivity (Laberge et al. 2016; Soliman et al. 2017). Owing to the physical impact of UFs, new research is recommended on treatment modalities and individualised care to minimise symptom burden and promote the well-being of affected individuals.

The study revealed that reduced or no sexual activity was a major concern for participants because of symptoms of UFs, leading to conflicts between couples. Similar findings such as painful sexual intercourse and unspecified negative impact on sexual life among women diagnosed with UFs were reported by other research studies (Hervé et al. 2018; Moshesh et al. 2014; Zimmermann et al. 2012). Our findings indicated that participants suffered infertility as a complication of UFs, which resulted in significant emotional and psychological challenges such as fear of rejection from spouses, depression related to childlessness and a perceived reduction in the fundamental value of womanhood. Findings from other similar studies investigating UFs experiences echoed that UFs reduce pregnancy and live birth rates depending on the location and size of UFs, distort the endometrial activity and have a damaging effect on fertility (Freytag et al. 2021; Ndububa 2016; Van Heertum & Barmat 2014; Zepiridis, Grimbizis & Tarlatzis 2016). In Eswatini, high value is placed on motherhood, a woman’s worth is measured by her ability to bear children (Nyawo 2021). As such women who cannot bear children are subjected to stigma, resulting in further psychological and emotional distress (Ofosu-Budu & Hanninen 2020). More research is needed to develop new treatment measures and promote male involvement in the care of women diagnosed with UFs.

The study found that participants experienced psychological effects from UFs, including depression, anxiety, stress, frustration and isolation. Uterine fibroids recurrence after myomectomy caused depression, physical symptoms of UFs caused stress, treatment options were frustrating and participants preferred isolation because of the symptoms of UFs. These findings tie in with findings from other research studies that identified the psychological impacts of UFs (Chiuve et al. 2022; Dykstra et al. 2023; Ghant et al. 2015). This indicates the need for mental and psychological management of UFs to assist the affected population in coping with the implications of UFs.

Most participants experienced a high emotional burden of UFs, including fear related to treatment options, rejection by spouses, fear of the disease outcome, worry and sadness. These findings are similar to other findings from previous studies that identified the emotional impact of UFs (Ghant et al. 2014, 2015; Knudsen et al. 2019). A holistic approach to the management of UFs should be emphasised including education and emotional counselling to allay fears and correct misconceptions as recommended by other studies (Elsaied et al. 2020; Odebode, Adegboyega & Yusuf 2015).

Most participants experienced a negative social impact because of UFs symptoms and related complications such as loss of marriage prospects because of infertility, poor relationships and mistrust between couples related to sexual disturbances. These findings are consistent with other studies identifying that UFs significantly impair psychosocial functioning (Ghant et al. 2015; Go et al. 2020). Social support should be included in individuals diagnosed with UFs to promote quality of life.

### Strengths

The design of this study allowed the researcher to interact with participants, observe their body language and make follow-up questions, ensuring that rich data were collected. New information on UFs was discovered as the participants discussed their lived experiences, and much more diverse perspectives of the disease were revealed. Findings from this study will increase the body of knowledge about the disease among women. These findings can also guide health professionals and policymakers to design congruent UFs management strategies to deal with the different experiences.

### Limitations

The research used the focus group discussions approach, so the subjective nature of this study could pose a limitation. It may be difficult to get honest opinions on sensitive topics as the majority opinions may influence some participants in the group. The study was done during the high prevalence of coronavirus disease 2019 (COVID-19), so forming the focus group was time consuming and costly because a special arrangement was made with hospital management and participants.

### Implications

The study revealed that women with UFs experience significant physical, emotional, psychological and social distress. Revised UFs clinical care strategies that include patient-centered care, male involvement and psychosocial management could decrease the disease burden and enhance individualised care. Raising public awareness and offering educational programmes to help women make informed treatment decisions are also vital. National-level disease awareness and early introduction of educational programmes on UFs in gynaecology clinics and communities can provide women with the right information, allay the emotional and psychological impact of the disease and promote positive health-seeking behaviour.

## Conclusion

The current study provided a wide range of lived experiences of women with UFs through in-depth focus group discussions, revealing complex physical, psychological, emotional and social challenges. Physical symptoms include lower abdominal pains and abnormal vaginal bleeding, while psychological and emotional issues include depression, stress and fear. Lack of disease awareness, beliefs and perceptions influenced their experience of symptoms and health-seeking behaviour. Future research is recommended to concentrate on developing educational programmes to educate women and communities, implementing psychological therapies and providing patient-centred care to safeguard their emotional well-being and curtail the disease impact. These findings can provide guidance and direction to practitioners and policymakers to develop collaborative and psychosocial management approaches for patients with UFs.
